# Risks of recurrent stroke and all serious vascular events after spontaneous intracerebral haemorrhage: pooled analyses of two population-based studies

**DOI:** 10.1016/S1474-4422(21)00075-2

**Published:** 2021-06

**Authors:** Linxin Li, Michael T C Poon, Neshika E Samarasekera, Luke A Perry, Tom J Moullaali, Mark A Rodrigues, James J M Loan, Jacqueline Stephen, Christine Lerpiniere, Maria A Tuna, Sergei A Gutnikov, Wilhelm Kuker, Louise E Silver, Rustam Al-Shahi Salman, Peter M Rothwell

**Affiliations:** a Wolfson Centre for Prevention of Stroke and Dementia, Nuffield Department of Clinical Neurosciences, University of Oxford, Oxford, UK; b Centre for Clinical Brain Sciences, University of Edinburgh, Edinburgh, UK; c Usher Institute, University of Edinburgh, Edinburgh, UK; d Centre for Discovery Brain Sciences, University of Edinburgh, Edinburgh, UK; e Department of Anaesthesia and Pain Management, Royal Melbourne Hospital, Parkville, VIC, Australia; f The George Institute for Global Health, Sydney, NSW, Australia; g Department of Neuroradiology, NHS Lothian, Edinburgh, UK

## Abstract

**Background:**

Patients with stroke due to spontaneous (non-traumatic) intracerebral haemorrhage (ICH) are at risk of recurrent ICH, ischaemic stroke, and other serious vascular events. We aimed to analyse these risks in population-based studies and compare them with the risks in RESTART, which assessed antiplatelet therapy after ICH.

**Methods:**

We pooled individual patient data from two prospective, population-based inception cohort studies of all patients with an incident firs-in-a-lifetime ICH in Oxfordshire, England (Oxford Vascular Study; April 1, 2002, to Sept 28, 2018) and Lothian, Scotland, UK (Lothian Audit of the Treatment of Cerebral Haemorrhage; June 1, 2010, to May 31, 2013). We quantified the absolute and relative risks of recurrent ICH, ischaemic stroke, or any serious vascular event (non-fatal stroke, non-fatal myocardial infarction, or vascular death), stratified by ICH location (lobar *vs* non-lobar) and comorbid atrial fibrillation (AF). We compared pooled event rates with those after allocation to avoid antiplatelet therapy in RESTART.

**Findings:**

Among 674 patients (mean age 74·7 years [SD 12·6], 320 [47%] men) with 1553 person-years of follow-up, 46 recurrent ICHs (event rate 3·2 per 100 patient-years, 95% CI 2·0–5·1) and 25 ischaemic strokes (1·7 per 100 patient-years, 0·8–3·3) were reported. Patients with lobar ICH (n=317) had higher risk of recurrent ICH (5·1 per 100 patient-years, 95% CI 3·6–7·2) than patients with non-lobar ICH (n=355; 1·8 per 100 patient-years, 1·0–3·3; hazard ratio [HR] 3·2, 95% CI 1·6–6·3; p=0·0010), but there was no evidence of a difference in the risk of ischaemic stroke (1·8 per 100 patient-years, 1·0–3·2, *vs* 1·6 per 100 patient-years, 0·6–4·4; HR 1·1, 95% CI 0·5–2·8). Conversely, there was no evidence of a difference in recurrent ICH rate in patients with AF (n=147; 3·3 per 100 patient-years, 95% CI 1·0–10·7) compared with those without (n=526; 3·2 per 100 patient-years, 2·2–4·7; HR 0·9, 95% CI 0·4–2·1), but the risk of ischaemic stroke was higher with AF (6·3 per 100 patient-years, 3·7–10·9, *vs* 0·7 per 100 patient-years, 0·1–5·6; HR 8·2, 3·3–20·3; p<0·0001), resulting in patients with AF having a higher risk of all serious vascular events than patients without AF (15·5 per 100 patient-years, 10·0–24·1, *vs* 6·8 per 100 patient-years, 3·6–12·5; HR 1·78, 95% CI 1·16–2·74; p=0·0090). Only for patients with lobar ICH without comorbid AF was the risk of recurrent ICH greater than the risk of ischaemic stroke (5·2 per 100 patient-years, 95% CI 3·6–7·5, *vs* 0·9 per 100 patient-years, 0·2–4·8; p=0·00034). Comparing data from the pooled population-based studies with that from patients allocated to not receive antiplatelet therapy in RESTART, there was no evidence of a difference in the rate of recurrent ICH (3·5 per 100 patient-years, 95% CI 1·9–6·0, *vs* 4·4 per 100 patient-years, 2·6–6·1) or ischaemic stroke (3·4 per 100 patient-years, 1·9–5·9, *vs* 5·3 per 100 patient-years, 3·3–7·2).

**Interpretation:**

The risks of recurrent ICH, ischaemic stroke, and all serious vascular events after ICH differ by ICH location and comorbid AF. These data enable risk stratification of patients in clinical practice and ongoing randomised trials.

**Funding:**

UK Medical Research Council, Stroke Association, British Heart Foundation, Wellcome Trust, and the National Institute for Health Research Oxford Biomedical Research Centre.

## Introduction

Worldwide, stroke due to spontaneous (non-traumatic) intracerebral haemorrhage (ICH) accounts for approximately a quarter of all strokes but almost half of the disability-adjusted life-years lost owing to stroke,^1^, ^2^ because of the subsequent risks of death, disability, and serious vascular events. Adults with ICH usually have underlying cerebral small vessel disease,^3^ which leaves them at risk of recurrent stroke^4^ and systemic comorbidities, which put them at additional risk of stroke and other cardiovascular events.^5^, ^6^

Overall, according to data from seven cohorts,^6^, ^7^, ^8^, ^9^ ICH survivors seem to have a similar annual risk of recurrent ICH (1·1–3·9%) and ischaemic stroke (1·1–3·2%). However, these estimates of the risk of stroke recurrence originate mostly from hospital-based studies. Although a recent study^8^ reported major ischaemic and haemorrhagic events after ICH in a cohort of 560 patients, little is known about the risks of all serious vascular events.

**Research in context**

**Evidence before this study**

We searched Ovid MEDLINE (from 1946), Embase (from 1976), and bibliographies of relevant publications on June 18, 2020 (appendix p 2), for cohort studies, published in English in full, of any serious vascular event after intracerebral haemorrhage (ICH) from database inception to June 18, 2020. We found 21 published studies. Mostly hospital-based cohort studies, with unavoidable selection biases, have described the risks and risk factors for selected outcomes after ICH over short durations of follow-up. The absolute event rate ranged between 1·1 and 11·6 per 100 patient-years for recurrent ICH and between 1·0 and 3·0 per 100 patient-years for ischaemic stroke. No study reported the risks of serious vascular events after ICH. Six studies reported the risks of recurrent ICH versus ischaemic stroke by ICH location, with conflicting results. No study compared risks by comorbid atrial fibrillation (AF). Recently, RESTART found that after ICH associated with antithrombotic drug use, survivors had a non-significantly lower risk of recurrent ICH after starting antiplatelet therapy compared with not receiving these drugs. However, the generalisability of RESTART to real-world practice is unknown.

**Added value of this study**

These pooled analyses provide data from two contemporary population-based cohort studies, free of selection bias, with prospective follow-up for not only recurrent stroke, but also for all serious vascular events after ICH. The rates of ischaemic stroke and recurrent ICH in our cohort studies support the generalisability of the event rates observed in RESTART. A meta-analysis of our cohort studies and another four similar published cohorts established lobar ICH as a risk factor for recurrent ICH, but not for ischaemic stroke after ICH. We have identified comorbid AF as the major risk factor for ischaemic stroke and all serious vascular events after ICH.

**Implications of all the available evidence**

In clinical practice, ICH survivors can be stratified into groups at higher risk of recurrent ICH, ischaemic stroke, or all serious vascular events according to ICH location and comorbid AF. The high risk of ischaemic stroke and all serious vascular events for ICH survivors with AF mandates rapid completion of ongoing randomised controlled trials of antithrombotic drugs or left atrial appendage occlusion to reduce these risks.

Identifying risk factors for recurrent ICH, ischaemic stroke, and all serious vascular events after ICH could help with risk stratification to inform decisions about antithrombotic drugs after ICH. Lobar ICH location has been associated with a higher risk of recurrent ICH in some studies,^5^, ^6^, ^8^, ^9^, ^10^, ^11^, ^12^ but not others.^13^, ^14^ The risk of recurrent stroke is particularly high in the first few days and weeks after transient ischaemic attack (TIA) or ischaemic stroke,^15^ although less is known about the time course of recurrent ICH. Some studies have found that the risks of recurrent ICH were particularly high in the first year after ICH,^16^, ^17^ although most studies only included 30-day survivors so they are likely to have underestimated the true early risk. Risk factors for ischaemic stroke after ICH are also unclear. Although ICH location does not seem to be associated with the risk of ischaemic stroke,^6^, ^8^, ^9^ atrial fibrillation (AF) is likely to be a risk factor for ischaemic stroke, but not recurrent ICH.^18^

In 2019, findings from RESTART^19^ showed that after ICH associated with antithrombotic drug use, survivors had a numerically but non-significantly lower risk of recurrent ICH after starting antiplatelet therapy compared with avoiding these drugs (adjusted hazard ratio [HR] 0·51, 95% CI 0·25–1·03; p=0·060). However, RESTART left some uncertainties. First, although there was no evidence of heterogeneity of the effects of antiplatelet therapy by ICH location in RESTART, participants with non-lobar ICH might have benefited more than people with lobar ICH.^19^, ^20^ Second, RESTART recruited one in 12 eligible patients and the average ICH volume was approximately 4 mL,^21^ so the generalisability of the trial's event rates needs to be established in comparison with population-based data from unselected patients with ICH.^22^ Third, there was no heterogeneity of the effects of antiplatelet therapy for ICH survivors with comorbid AF, but if these patients have especially high risks of systemic embolism then oral anticoagulation might be warranted.^23^

Therefore, we analysed two contemporary, prospective, population-based cohort studies in the UK to address three uncertainties: first, the absolute and relative risks of recurrent ICH and ischaemic stroke, stratified by ICH location and comorbid AF, in unselected patients with ICH; second, the risks of all serious vascular events after ICH; and third, the generalisability of RESTART.

## Methods

### Cohort studies

The Oxford Vascular Study (OXVASC) is an ongoing population-based inception cohort study of all acute vascular events in a population of 92 728 individuals, registered with 100 general practitioners in nine general practices in Oxfordshire, UK.^24^ OXVASC used multiple overlapping methods to achieve near-complete ascertainment of all cases:^24^ (1) a daily, rapid-access clinic to which participating general practitioners and the local emergency department team referred individuals with suspected TIA or minor stroke; (2) daily searches of admissions to medical, stroke, neurology, and other relevant wards; (3) daily searches of the local emergency department attendance register; (4) daily searches of in-hospital death records via the bereavement office; (5) monthly searches of all death certificates and coroner's reports for out-of-hospital deaths; (6) monthly searches of general practitioner diagnostic coding and hospital discharge codes; and (7) monthly searches of brain and vascular imaging referrals. We included patients diagnosed with ICH by brain imaging or pathological examination between April 1, 2002, and Sept 28, 2018, inclusive.

The Lothian Audit of the Treatment of Cerebral Haemorrhage (LATCH) is an ongoing population-based audit and inception cohort study of adults aged at least 16 years with ICH in the National Health Service (NHS) Lothian health board region of Scotland, UK (mid-2012 population 843 733).^5^ LATCH also used multiple overlapping methods to achieve near-complete ascertainment of data: (1) notifications from a collaborative Lothian-wide network of physicians, neurologists, neurosurgeons, radiologists, pathologists, stroke specialist nurses, and stroke audit personnel; (2) daily review of all brain imaging; (3) quarterly searches of the electronic patient records system in secondary care; (4) annual searches of death certificates and coroner's reports for sudden deaths; and (5) annual searches of NHS Lothian records in the Scottish Stroke Care Audit. We included patients diagnosed with ICH by brain imaging or pathological examination between June 1, 2010, and May 31, 2013, inclusive.

Written informed consent or assent from relatives was obtained from all participants in OXVASC, which was approved by the local research ethics committee (OREC A: 05/Q1604/70). LATCH was approved by the NHS Lothian Caldicott Guardian on the basis that patients in NHS Lothian were informed about the use of their data for audit by information leaflets, which informed patients and their carers about their right to opt out; these analyses of an anonymised extract of data did not require research ethics committee approval. We planned the pooling, outcomes, and analyses of the cohorts prospectively, after they were presented simultaneously at the European Stroke Organisation conference in 2019.

In both studies, demographic data, vascular risk factors, and medication used before ICH were collected from medical records or face-to-face interviews and cross-referenced with primary care records. Brain CT imaging was the first-line imaging method used for patients with stroke in both studies (unless presentation was delayed, in which case brain MRI was used). A dedicated study neuroradiologist reviewed brain imaging centrally to confirm ICH and categorised ICH location as lobar or non-lobar (involving the basal ganglia, thalamus, internal or external capsule, brainstem, or cerebellum). In LATCH, the Cerebral Haemorrhage Anatomical Rating Instrument (CHARTS) was used and in OXVASC the principles used were broadly similar to the subsequently published CHARTS.^25^ Selected cases were further investigated for underlying causes using CT or magnetic resonance (MR) angiography, CT or MR venography, or catheter angiography or brain MRI, especially in people younger than 50 years, in the presence of signs suggestive of an underlying structural cause,^26^ or in the absence of other risk factors.^27^ For the current analysis, we included consecutive patients with first ever ICH without evidence of it being secondary to trauma, thrombolysis, haemorrhagic transformation of infarction, or underlying macrovascular or neoplastic causes. We excluded adults with exclusively extra-axial intracranial haemorrhage.

Patients' physicians usually stopped premorbid antithrombotic treatment immediately after ICH diagnosis, and the decision to restart an antithrombotic drug was based on physicians' clinical judgment of the risk and benefit for each patient.

In OXVASC,^24^ patients were followed up face-to-face at 1, 6, 12, 60, and 120 months by a study nurse or physician supplemented by review of primary care records. OXVASC personnel followed up patients who had moved out of the study area by telephone at the same timepoints as face-to-face follow-up. In LATCH,^5^ patients were followed up annually using postal or telephone questionnaires sent to each adult's general practitioner to ascertain vital status and the occurrence of any outcome events. LATCH personnel recorded outcomes and causes of deaths during follow-up according to clinical information obtained by direct follow-up, via primary care records, or by record linkage to hospital admissions and death records.

### Outcomes

The primary outcomes in this study were recurrent ICH, ischaemic stroke, or any serious vascular event (non-fatal stroke, non-fatal myocardial infarction, or vascular death).^28^, ^29^ We defined vascular death as death within 30 days following and due to recurrent symptomatic ICH, extracranial haemorrhage, ischaemic stroke, myocardial infarction, peripheral artery occlusion, mesenteric ischaemia, central retinal arterial occlusion, symptomatic deep vein thrombosis, symptomatic pulmonary embolism, sudden cardiac death (with symptoms suggestive of myocardial infarction or evidence of arrhythmia), symptomatic stroke of uncertain subtype, or revascularisation procedures.^19^

### Systematic review and meta-analysis

LL searched Ovid MEDLINE (from 1946) and Embase (from 1974) using the terms detailed in the appendix (p 2) for any ICH cohort study reporting absolute event rates (or total person-years of follow-up and numbers of outcomes) of ischaemic stroke or recurrent ICH stratified by ICH location (lobar *vs* non-lobar) that were published in English in full (ie, not just as a conference abstract) up to June 18, 2020. We excluded studies that were restricted to a selected ICH subgroup (eg, ICH attributed to hypertension alone, cerebral amyloid angiopathy, or antithrombotic treatment), that included 50 patients or fewer, or had an average follow-up of 1 year or less. LL and MTCP identified eligible studies independently, consulted a third reviewer (PMR) in case of disagreement, and screened bibliographies of included studies for additional studies. We extracted information on the population studied, study period (duration), sample size, person-years of follow-up, and total numbers of recurrent ICH and ischaemic strokes stratified by index ICH location. We calculated the risk ratio of ischaemic stroke or recurrent ICH after lobar versus non-lobar ICH in OXVASC and LATCH and in included cohort studies, and then pooled the risk ratios using a random effects model with inverse variance weighting; we used the *I*^2^ statistic to measure heterogeneity between studies.

### Statistical analysis

We harmonised the classification and coding of categorical covariates in the two cohorts, analysed each cohort separately, and then did the same analyses using a pooled dataset of all individual patient-level data from both cohorts. We did complete analyses without imputation and report missing data where applicable. We compared baseline characteristics of patients with versus without the candidate risk factors (lobar *vs* non-lobar ICH and presence *vs* absence of comorbid AF [defined as any known history of AF or new AF at the time of ICH presentation]) with the χ^2^ test for categorical variables and *t* test for continuous variables.

We calculated absolute event rates for the primary outcomes separately for each cohort. We then calculated pooled estimates using an inverse variance weighted random effect meta-analysis to account for potential heterogeneity between the two cohorts. We derived figures for the cumulative incidence of outcomes from Kaplan-Meier analyses until the occurrence of the first outcome during follow-up or censoring at death, last available follow-up, or Sept 28, 2018, in OXVASC or Feb 14, 2018, in LATCH. Owing to the differences in follow-up duration between the two studies, we also did sensitivity analyses using follow-up data censored at 5 years when pooling both studies.

We applied Poisson regression models using the number of events as the outcome, including the person-years at risk as an offset to estimate the absolute event rates with 95% CIs (using all follow-up and also censoring at 5 years in sensitivity analyses). We used unadjusted Cox regression models to compare the risk of the first occurrence of each outcome (ischaemic stroke, recurrent ICH, or any serious vascular event) during follow-up to 5 years according to the candidate risk factors (ICH location and comorbid AF). We also did Cox regression adjusting for study clustering using different approaches including a stratified Cox regression, a two-stage random effects meta-analysis, and a Cox regression model with the cohort as a covariate. In view of the potential competing risk of death, we also did sensitivity analyses using competing risk models (Fine-Gray sub-distribution hazard model).^30^ We did additional sensitivity analyses stratified by use of antithrombotic therapy before ICH.

In the subgroup of patients who had used antithrombotic therapy until ICH, we compared the risks of ischaemic stroke and recurrent ICH in the two population-based cohort studies with the same risks for the participants allocated to avoid antithrombotic therapy in RESTART.

All analyses were done in SPSS version 22 or Stata 15.

### Role of the funding source

The funders of the study had no role in study design, data collection, data analysis, data interpretation, or writing of the report.

## Results

674 patients (mean age 74·7 years [SD 12·6], 320 [47%] men; table 1) presented with incident first-ever ICH (419 in LATCH and 255 in OXVASC), of whom 317 (47%) had lobar ICH, 355 (53%) had non-lobar ICH, and two (<1%) had fatal ICH that occurred out of the study area without accessible details of the ICH location. These two patients were not included in the analyses stratified by ICH location. The cohorts were similar at baseline, apart from significantly higher frequencies of smoking and use of antiplatelet drugs and statins before ICH in LATCH, and a higher frequency of antihypertensive drug use at hospital or clinic discharge in OXVASC (appendix p 3).

**Table 1:** Characteristics of all patients with first-ever ICH at diagnosis and hospital discharge, stratified by ICH location

	Total (n=674)*	**Lobar ICH (n=317)**	**Non-lobar ICH (n=355)**	**p value**
**Presentation**				
Age, years	74·7 (12·6)	75·5 (11·6)	74·1 (13·5)	0·16
**Sex**				
Male	320 (47%)	139 (44%)	181 (51%)	0·064
Female	354 (53%)	178 (56%)	174 (49%)	..
**Medical history**				
Previous occlusive vascular disease†	217/673 (32%)	103/317 (32%)	112/354 (32%)	0·81
Hypertension	429/673 (64%)	178/317 (56%)	250/354 (71%)	<0·0001
Diabetes	83/674 (12%)	37/317 (12%)	46/355 (13%)	0·61
Hyperlipidaemia	134/673 (20%)	58/317 (18%)	76/354 (21%)	0·31
Atrial fibrillation	147/673 (22%)	63/317 (20%)	84/354 (24%)	0·23
Current smoker	116/665 (17%)	54/311 (17%)	62/352 (18%)	0·93
**Medication before ICH**				
Antithrombotic drugs‡	344/674 (51%)	172/317 (54%)	171/355 (48%)	0·12
Anticoagulant drugs	110/674 (16%)	52/317 (16%)	58/355 (16%)	0·98
Antiplatelet drugs	250/674 (37%)	128/317 (40%)	121/355 (34%)	0·092
Antihypertensive drugs	329/674 (49%)	142/317 (45%)	186/355 (52%)	0·049
Statins	222/644 (34%)	117/304 (38%)	104/338 (31%)	0·040
**Medication at hospital or clinic discharge**§				
Antithrombotics¶	22/378 (6%)	12/182 (7%)	10/196 (5%)	0·54
Anticoagulant drugs	7/378 (2%)	5/182 (3%)	2/196 (1%)	0·21
Antiplatelet drugs	16/378 (4%)	8/182 (4%)	8/196 (4%)	0·88
Antihypertensive drugs	229/378 (61%)	89/182 (49%)	140/196 (71%)	<0·0001

Data are mean (SD), number (%), or n/N (%). Missing data in Lothian Audit of the Treatment of Cerebral Haemorrhage: previous occlusive vascular disease (n=1), hypertension (n=1), hyperlipidaemia (n=1), atrial fibrillation (n=1), smoking (n=2), and statins (n=30). Missing data in Oxford Vascular Study: smoking (n=8). ICH=intracerebral haemorrhage.

* Two patients with unknown location owing to out-of-area death and brain imaging not accessible.

† Any history of transient ischaemic attack, stroke, myocardial infarction, or peripheral vascular disease.

‡ 16 patients were on both anticoagulant and antiplatelet drugs.

§ Excluding patients who died before discharge.

¶ One patient was on both anticoagulant and antiplatelet drugs.

Patients with lobar ICH were less likely than patients with non-lobar ICH to have been diagnosed with hypertension (178 [56%] of 317 *vs* 250 [71%] of 354; p<0·0001) and to have taken blood pressure lowering drugs both before ICH (142 [45%] of 317 *vs* 186 [52%] of 355; p=0·049) and at discharge from hospital (89 [49%] of 182 *vs* 140 [71%] of 196; p<0·0001), whereas patients with lobar ICH were more likely than patients with non-lobar ICH to have taken a statin before ICH (117 [38%] of 304 *vs* 104 [31%] of 338; p=0·040), but there were no other baseline differences by ICH location (table 1). Baseline characteristics by ICH location were broadly similar in analyses of each cohort (appendix pp 4, 5).

147 (22%) of 673 patients had comorbid AF at presentation with ICH; they were older, more likely to have peripheral vascular disease and hypertension, and more likely to have used oral anticoagulation and blood pressure lowering drugs before ICH than those without AF (appendix p 6).

Before ICH, 344 (51%) of 674 patients were taking antithrombotic therapy, which was continued by only 22 (6%) of 378 survivors at discharge from hospital or clinic, with no significant differences by ICH location (table 1) or study cohort (appendix pp 3–5). Although patients with AF were more likely to be on antithrombotic drug therapy at hospital discharge, only five (6%) of 84 ICH survivors with comorbid AF took oral anticoagulation at hospital or clinic discharge (appendix p 6).

During 1553 patient-years of follow-up, 492 deaths, 46 recurrent ICHs, 25 ischaemic strokes, and 118 serious vascular events were reported. The details of the recurrent ICH and ischaemic strokes are presented in the appendix (p 7). Overall, the absolute event rate of recurrent ICH (3·2 per 100 patient-years, 95% CI 2·0–5·1) was greater than the rate of ischaemic stroke (1·7 per 100 patient-years, 0·8–3·3; p=0·010); the absolute event rate of any serious vascular event was 7·9 per 100 patient-years (95% CI 4·3–14·4; table 2). These risks were similar in sensitivity analyses censoring follow-up at 5 years (appendix p 8).

**Table 2:** Absolute event rates of first recurrent ICH, ischaemic stroke, or any serious vascular event in patients with ICH stratified by ICH location and history of AF

		**Recurrent ICH**		**Ischaemic stroke**		Serious vascular event*	
		Number of events/patient-years	Event rate per 100 patient-years (95% CI)	Number of events/patient-years	Event rate per 100 patient-years (95% CI)	Number of events/patient-years	Event rate per 100 patient-years (95% CI)
**All**							
OXVASC (n=255)†		15/626	2·4 (1·3–4·0)	7/637	1·1 (0·4–2·3)	35/616	5·7 (4·0–7·9)
LATCH (n=419)		31/788	3·9 (2·7–5·6)	18/801	2·3 (1·3–3·6)	83/785	10·6 (8·4–13·1)
Pooled		46/1414	3·2 (2·0–5·1)	25/1438	1·7 (0·8–3·3)	118/1401	7·9 (4·3–14·4)
**ICH location**							
OXVASC							
	Lobar (n=109)	11/275	4·0 (2·7–7·2)	4/275	1·5 (0·4–3·7)	23/271	8·5 (5·4–12·7)
	Non-lobar (n=144)	4/351	1·1 (0·3–2·9)	3/362	0·8 (0·2–2·4)	12/345	3·5 (1·8–6·1)
LATCH							
	Lobar (n=208)	22/384	5·7 (3·6–8·7)	8/388	2·1 (0·9–4·1)	42/384	10·9 (7·9–14·8)
	Non-lobar (n=211)	9/404	2·2 (1·0–4·2)	10/412	2·4 (1·2–4·5)	41/401	10·2 (7·3–13·9)
Pooled							
	Lobar (n=317)	33/659	5·1 (3·6–7·2)	12/664	1·8 (1·0–3·2)	65/656	10·0 (7·8–12·8)
	Non-lobar (n=355)	13/755	1·8 (1·0–3·3)	13/774	1·6 (0·6–4·4)	53/745	6·1 (2·1–17·6)
**Comorbid AF**							
OXVASC							
	AF (n=57)	1/81	1·2 (0·0–6·9)	6/81	7·4 (2·7–16·1)	9/79	11·5 (5·2–21·8)
	No AF (n=198)	14/545	2·6 (1·4–4·3)	1/556	0·2 (0·0–1·0)	26/537	4·8 (3·2–7·1)
LATCH							
	AF (n=90)	6/126	4·8 (1·8–10·4)	7/127	5·5 (2·2–11·4)	23/126	18·2 (11·6–27·4)
	No AF (n=328)	25/662	3·8 (2·4–5·6)	11/674	1·6 (0·8–2·9)	60/659	9·1 (7·0–11·7)
Pooled							
	AF (n=147)	7/207	3·3 (1·0–10·7)	13/208	6·3 (3·7–10·9)	32/205	15·5 (10·0–24·1)
	No AF (n=526)	39/1207	3·2 (2·2–4·7)	12/1230	0·7 (0·1–5·6)	86/1196	6·8 (3·6–12·5)

AF=atrial fibrillation. ICH=intracerebral haemorrhage. LATCH=Lothian Audit of the Treatment of Cerebral Haemorrhage. OXVASC=Oxford Vascular Study.

* Non-fatal stroke or myocardial infarction, or vascular death.

† Cause of death data missing for three patients because patients moved out of the area or abroad.

**Figure 1:**
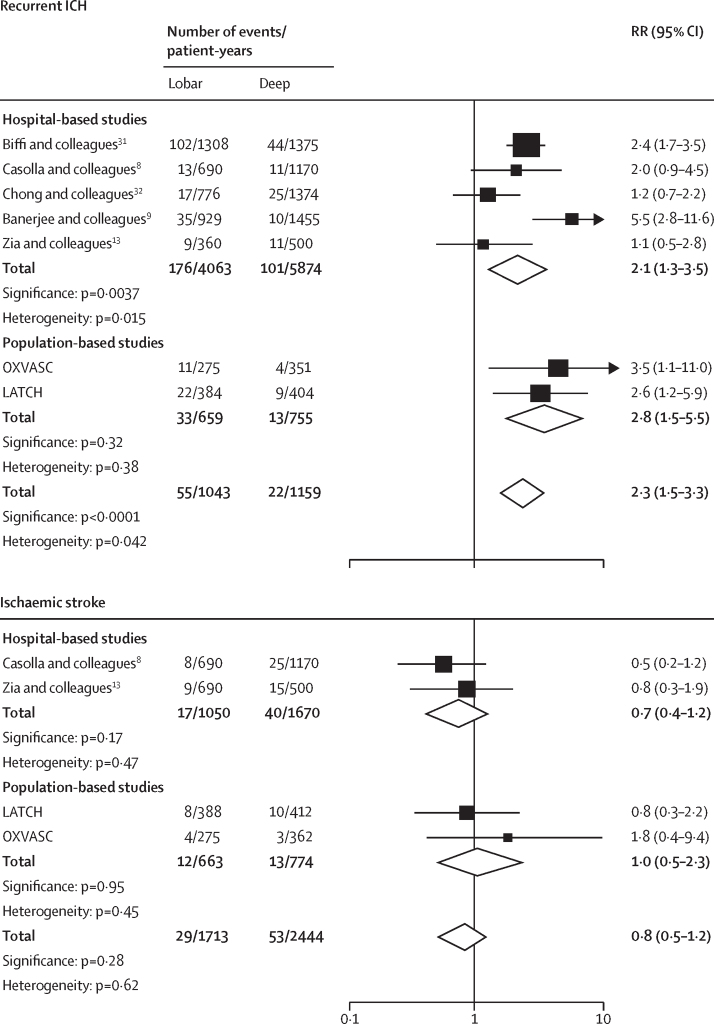
Pooled analyses of the relative risks of recurrent ICH and ischaemic stroke following lobar ICH versus non-lobar ICH ICH=intracerebral haemorrhage. LATCH=Lothian Audit of the Treatment of Cerebral Haemorrhage. OXVASC=Oxford Vascular Study. RR=rate ratio.

**Figure 2:**
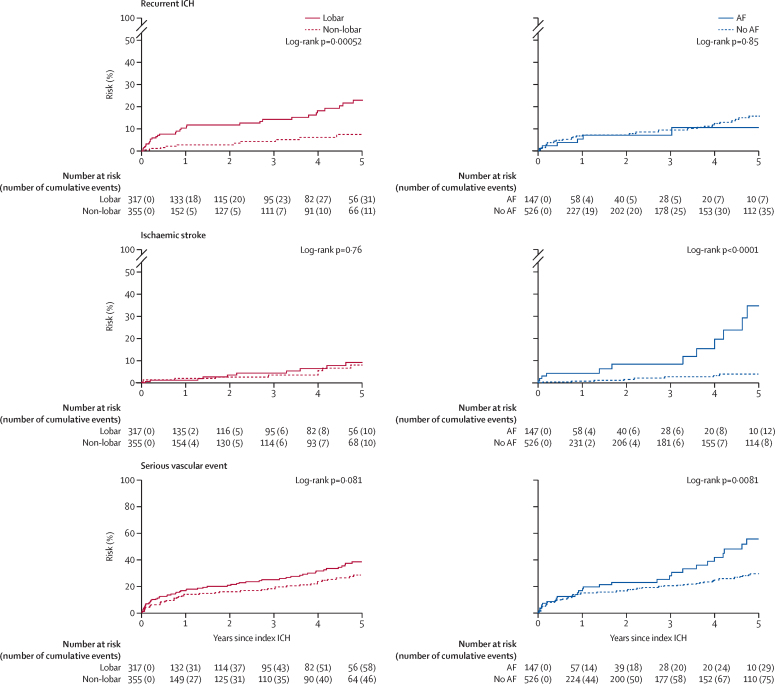
5-year risks of first recurrent ICH, ischaemic stroke, or serious vascular event stratified by ICH location and by AF in pooled analyses of OXVASC and LATCH Serious vascular events were non-fatal stroke or myocardial infarction, or vascular death. AF=atrial fibrillation. ICH=intracerebral haemorrhage. LATCH=Lothian Audit of the Treatment of Cerebral Haemorrhage. OXVASC=Oxford Vascular Study.

The absolute event rate of recurrent ICH was higher after lobar (5·1 per 100 patient-years, 95% CI 3·6–7·2) versus non-lobar ICH (1·8 per 100 patient-years, 1·0–3·3; HR 3·2, 95% CI 1·6–6·3; p=0·0010; table 2). However, there was no evidence that the risk of ischaemic stroke differed by ICH location (lobar 1·8 per 100 patient-years, 95% CI 1·0–3·2 *vs* non-lobar 1·6 per 100 patient-years, 0·6–4·4; HR 1·1, 95% CI 0·5–2·8; p=0·76). The absolute event rate of recurrent ICH (5·1 per 100 patient-years, 95% CI 3·6–7·2) exceeded the risk of ischaemic stroke after lobar ICH (1·8 per 100 patient-years, 1·0–3·2). Finally, the absolute event rate of any serious vascular event was numerically greater after lobar (10·0 per 100 patient-years, 95% CI 7·8–12·8) than non-lobar ICH (6·1 per 100 patient-years, 2·1–17·6; HR 1·4, 95% CI 1·0–2·1; p=0·08). These findings were similar in separate analyses of each cohort (table 2); in sensitivity analyses censoring follow-up at 5 years (appendix p 8); after adjusting for clustering (appendix p 9); after using competing risk models (appendix pp 10, 11); in a meta-analysis of LATCH, OXVASC, and another five cohort studies that reported these risks stratified by ICH location (appendix pp 12, 13; figure 1);^8^, ^9^, ^13^, ^31^, ^32^ and in pooled analyses stratified by antithrombotic drug use before ICH (appendix p 14). Furthermore, pooled analyses of the time course of events in LATCH and OXVASC showed that the higher risk of recurrent ICH after lobar ICH (HR 3·2, 95% CI 1·6–6·3; p=0·0010) was most marked in the 90 days after ICH (6·0, 1·3–27·2; p=0·019; figure 2).

Comorbid AF conferred a higher risk of ischaemic stroke (AF 6·3 per 100 patient-years, 95% CI 3·7–10·9, *vs* no AF 0·7 per 100 patient-years, 0·1–5·6; HR 8·2, 95% CI 3·3–20·3; p<0·0001) and any serious vascular event (15·5 per 100 patient-years, 10·0–24·1, *vs* 6·8 per 100 patient-years, 3·6–12·5; HR 1·78, 95% CI 1·16–2·74; p=0·0090), but comorbid AF did not modify the risk of recurrent ICH (3·3 per 100 patient-years, 1·0–10·7, *vs* 3·2 per 100 patient-years, 2·2–4·7; HR 0·9, 95% CI 0·4–2·1; table 2; figure 2). 13 (52%) of the 25 ischaemic strokes were AF related, but only seven (15%) of the 46 patients with recurrent ICH had AF. The associations with comorbid AF were similar in separate analyses of each cohort (table 2), in sensitivity analyses censoring follow-up at 5 years (appendix p 8), after adjusting for clustering (appendix p 9), and after using a competing risk model (appendix p 15). Stratification of the cohort according to presence versus absence of both comorbid AF or occlusive vascular disease before ICH confirmed no association between comorbid AF and recurrent ICH in all four groups, but the absolute event rate of any serious vascular event after ICH and comorbid AF alone (11·3 per 100 patient-years, 95% CI 6·3–20·4) was numerically higher when accompanied by a history of occlusive vascular disease (19·4 per 100 patient-years, 95% CI 9·0–41·7; appendix p 16). Findings were similar in an analysis censoring at 5-year follow-up (appendix p 17).

Considering both lobar ICH location and comorbid AF, there was no evidence that the absolute event rates of recurrent ICH and ischaemic stroke differed, apart from patients with lobar ICH and no history of AF for whom the risk of recurrent ICH was greater than the risk of ischaemic stroke (5·2 per 100 patient-years, 95% CI 3·6–7·5, *vs* 0·9, 0·2–4·8; p=0·00034; appendix p 18). Findings were similar in an analysis censoring at 5-year follow-up (appendix p 19). Nonetheless, in keeping with the high risk for all serious vascular events in patients with comorbid AF (table 2), the risk of any serious vascular event was numerically higher for patients with lobar ICH and comorbid AF (14·6 per 100 patient-years, 95% CI 8·6–24·6) than for patients with lobar ICH in sinus rhythm (9·1 per 100 patient-years, 6·6–12·6; HR 1·3, 95% CI 0·7–2·5; p=0·42; appendix p 18). Findings were similar in an analysis censoring at 5-year follow-up (appendix p 19).

In the pooled cohorts, 344 (51%) of 674 patients were taking antithrombotic therapy before ICH (table 1). There was no evidence that the risk of recurrent ICH differed by use of antithrombotic therapy before ICH (yes *vs* no; HR 1·2, 95% CI 0·6–2·1), but the risk of ischaemic stroke was higher in patients who were taking antithrombotic therapy before ICH than those who were not (3·0, 1·2–7·5; p=0·020). Results were also consistent in analyses adjusting for clustering (appendix p 9). These patients might benefit from antithrombotic therapy after ICH, but 356 (94%) of 378 ICH survivors were not on antithrombotic drugs at hospital or clinic discharge. Therefore, we compared patients in the pooled cohorts who had taken antithrombotic therapy before ICH and met the eligibility criteria for the RESTART trial^19^ with patients who were allocated to not receive antiplatelet therapy in the RESTART trial to establish the external validity of RESTART (table 3; appendix p 20). Patients with ICH in the cohort studies were older and were more likely to have non-lobar ICH and comorbid AF in comparison with the RESTART participants, but the absolute event rate of recurrent ICH and ischaemic stroke did not differ (table 3). These findings were consistent in sensitivity analyses restricted to each cohort and when restricting analyses to 5 years of follow-up (appendix pp 21, 22). When using ICH location to stratify comparisons between the pooled cohorts and allocation to not receive antiplatelet therapy in RESTART, we did not find differences in the absolute event rate of ischaemic stroke and recurrent ICH, apart from a higher risk of recurrent ICH after non-lobar ICH in RESTART (5·5 per 100 patient-years, 95% CI 2·5–8·5) compared with the cohort studies (1·1 per 100 patient-years, 0·1–3·9; p=0·018; appendix p 23). Findings were similar when using the 5-year follow-up data (appendix p 24).

**Table 3:** Comparison of RESTART and the pooled cohort of OXVASC and LATCH using all follow-up data

		**RESTART (all; n=537)**	**RESTART (without antiplatelet therapy; n=269)**	Pooled OXVASC plus LATCH (fulfilling RESTART eligibility criteria;*n=246)	**p value**
**Baseline characteristics**					
Mean age, years		76	76	79	0·00010
Sex					
	Male	360 (67%)	187 (70%)	118 (48%)	<0·0001
	Female	177 (33%)	82 (30%)	128 (52%)	..
Previous hypertension		401 (75%)	207 (77%)	198 (80%)	0·33
Previous diabetes		127 (24%)	70 (26%)	42 (17%)	0·013
Previous atrial fibrillation		92 (17%)	50 (19%)	130 (53%)	<0·0001
Lobar ICH location		332 (62%)	166 (62%)	116 (47%)†	0·00094
**Risk of outcomes**					
Recurrent ICH					
	Number	35	23	13	..
	Rate (95% CI), per 100 patient-years	3·3 (2·2–4·4)†	4·4 (2·6–6·1)	3·5 (1·9–6·0)	0·52
Ischaemic stroke					
	Number	46	27	13	..
	Rate (95% CI), per 100 patient-years	4·4 (3·2–5·7)	5·3 (3·3–7·2)	3·4 (1·9–5·9)	0·19

ICH=intracerebral haemorrhage. OXVASC=Oxford Vascular Study. LATCH=Lothian Audit of the Treatment of Cerebral Haemorrhage.

* Patients in OXVASC and LATCH taking antithrombotic drugs before ICH for atrial fibrillation, previous transient ischaemic attack, ischaemic stroke, stroke of unknown subtype, peripheral artery disease, or myocardial infarction.

† Data missing for one patient.

## Discussion

In pooled analyses of 674 patients with first-ever ICH in two recent, prospective, population-based cohort studies, we found similar overall absolute event rates of recurrent ICH and ischaemic stroke and a high absolute event rate of any serious vascular event. Lobar ICH location was the principal risk factor for recurrent ICH. Comorbid AF was the principal risk factor for ischaemic stroke and all serious vascular events; the risks of these events were even greater in patients with a history of occlusive vascular disease. The risk of recurrent ICH was greater than the risk of ischaemic stroke only for patients with lobar ICH without comorbid AF. The risk of any serious vascular event was greater for patients with lobar ICH who had comorbid AF compared with patients in sinus rhythm. Participants allocated to not receive antiplatelet therapy in RESTART had similar absolute event rates of recurrent ICH and ischaemic stroke to those for the patients in the real-world pooled population-based studies who fulfilled eligibility criteria for RESTART.

Although hospital-based studies have not been consistent in identifying lobar ICH location as a risk factor for recurrent ICH, this was a consistent risk factor in our population-based studies and in a meta-analysis of all studies with the required data. One explanation for this finding is that moderate-to-severe cerebral amyloid angiopathy—which is a bleeding-prone vasculopathy that portends a particularly high risk of recurrent ICH in patients with imaging biomarkers of this disease^4^—underlies 58% of lobar ICHs, with 72% of patients also having moderate-to-severe small vessel disease.^3^ Another explanation is that only 49% of lobar ICH survivors took antihypertensive therapy at hospital discharge compared with 71% of non-lobar ICH; in the PROGRESS trial, blood pressure lowering reduced the risk of recurrent ICH by 49%,^33^ but use of antihypertensive treatment and adequate reduction of blood pressure are not always achieved in clinical practice, as reported by others.^31^, ^34^ These explanations likely contributed to the risk of recurrent ICH being highest early after lobar ICH.^8^, ^16^ Despite the difference in prevalence and treatment of high blood pressure by ICH location, lobar ICH location did not influence the risk of ischaemic stroke, probably because risk factors for occlusive vascular disease did not differ by ICH location.

Comorbid AF was associated with a more than eight times increased risk of ischaemic stroke and a doubling of the risk of any serious vascular event. This finding is consistent with expectations from studies of patients with AF, but no history of ICH. The CHA_2_DS_2_-VASc score is used for risk stratification of patients with AF, but its performance seems poor when applied to patients with ICH,^35^ although we found that risk stratification by history of occlusive vascular disease alone might be promising.

Considering these two risk factors together, we found that the risk of recurrent ICH was greater than the risk of ischaemic stroke solely in patients with lobar ICH and no history of AF (38% of all patients with ICH). However, the risk of any serious vascular event remained higher for patients with comorbid AF than for those in sinus rhythm after both lobar and non-lobar ICH. We found that 6% of ICH survivors started antithrombotic drugs at hospital discharge, which is lower than in a previous multicentre study,^36^ and the event rate of ischaemic stroke was 6·3%, which is similar to estimates from the placebo arms of the early prevention trials in patients with AF, but without ICH.^37^, ^38^ Therefore, we speculate that the priority for patients with lobar ICH alone remains reduction of the risk of recurrent ICH (by blood pressure lowering and the search for specific interventions for cerebral amyloid angiopathy),^39^ whereas for other patients—especially those with comorbid AF—the priority seems to be reduction of all serious vascular events with blood pressure lowering and antithrombotic drugs.

These real-world population-based studies provided some support for the external validity of RESTART. Although patients in our population-based studies were older, more likely to be female, and had a higher prevalence of comorbid AF with larger volumes of ICH compared with participants in RESTART, the overall risks of recurrent ICH and ischaemic stroke were comparable with the risks experienced by participants allocated to not receive antiplatelet therapy in RESTART. The numerically higher risk of ischaemic stroke in RESTART is perhaps explained by the fact that the decision to enrol patients in the trial was dependent on clinical equipoise and that RESTART investigators might have tended to include patients with higher risks of ischaemic stroke for whom the benefits of antiplatelet drugs were judged to be greater. The risk of recurrent ICH after non-lobar ICH was higher in RESTART than in our pooled cohorts, perhaps reflecting the inclusion of only high-risk patients with non-lobar ICH in RESTART (ie, clinicians might feel more confident treating patients with non-lobar ICH at lower risk of recurrence in everyday clinical practice); these differences do not appear to be attributable to the effects of antiplatelet therapy in RESTART, which might have reduced the risk of recurrent ICH after non-lobar ICH (adjusted HR 0·31, 95% CI 0·10–0·96).^19^

The strengths of our study include the pooling of individual patient-level data from two contemporaneous population-based studies, with a consistent inception point at the time of first-ever ICH, and prospective long-term follow-up. Our findings were consistent in various stratified analyses and sensitivity analyses using different methods and timeframes. But our study also has limitations. First, brain CT was the imaging method used in all patients, so we focused on ICH location, which was rated for all patients; we did not investigate risks according to MRI biomarkers of cerebral small vessel disease, which were available in a minority (<5%) in these real-world settings. Risks of recurrent ICH can be stratified according to imaging biomarkers of cerebral small vessel disease, by MRI^4^ or possibly CT,^3^ which would require much larger pooled analyses of cohort studies to further stratify absolute and relative risks of recurrent ICH and ischaemic stroke. Second, despite pooling two population-based studies, we did not have the power to explore interactions between ICH location, comorbid AF, and previous occlusive vascular disease. Third, although both cohorts were similar in the directions and magnitudes of the associations with risk factors of interest, the absolute risks of outcome events were higher in LATCH than OXVASC, probably owing to differences that we found in the prevalence of risk factors for vascular disease and in the uptake of secondary prevention drugs before ICH, as well as the known higher burden of cardiovascular disease in Scotland compared with England.^40^ However, the random effects model allowed us to take into account differences between cohorts. We did not adjust our estimates for the use of antithrombotic drugs after hospital or clinic discharge because so few patients started these drugs, and observational studies are not reliable for the assessment of treatment effects. Fourth, owing to their distinct mechanism and prognosis, extra-axial haemorrhages were not included in our study, and more data are needed to address how best to tackle the balance of recurrent bleeding and ischaemic stroke in these patients. Fifth, we focused on three risk factors for the outcomes of interest, but we did not analyse other factors such as patient disposition, functional status, and frailty. Finally, our results are based on a predominantly white population^5^, ^24^ and might not be generalisable to other countries, especially Asian populations for which the pattern of recurrence might differ.^14^

Our findings have implications for clinical practice and future research. Lobar ICH location can be used to stratify patients according to their risk of recurrent ICH, and comorbid AF can be used to stratify risk of ischaemic stroke and all serious vascular events after ICH. Given the apparent benefit of blood pressure lowering in reducing the risk of recurrent lobar ICH,^41^ the high frequency of recurrent ICH after lobar ICH should encourage greater use of blood pressure lowering therapy. Further research is needed to achieve larger sample sizes to explore risk stratification with greater precision and develop prognostic models using the three risk factors combined (ICH location, comorbid AF, and history of occlusive vascular disease) and other risk factors of interest (eg, biomarkers of cerebral small vessel disease). The high risk of all serious vascular events after ICH, whether ischaemic or haemorrhagic, and the poor uptake of blood pressure lowering therapy mandate more intensive approaches to secondary prevention; these include randomised trials of monitoring or therapeutic strategies to reduce blood pressure (NCT02699645 and NCT03863665), and a large randomised trial to investigate the reproducibility of the potentially beneficial effects of antiplatelet therapy on recurrent ICH and all serious vascular events seen in RESTART (NCT04522102). We found that for patients who stopped antithrombotic drugs after ICH or for those with known AF, the risk of ischaemic stroke was similar to the risk of recurrent ICH in those with lobar ICH, supporting the inclusion of these patients in recent and ongoing trials of starting versus stopping previous antithrombotic treatment in these subgroups (NCT03996772, NCT03950076, NCT02565693, NCT03186729, NCT03243175, NCT03153150).^19^ The high risk of all serious vascular events, which exceed the risk of recurrent ICH, for ICH survivors with comorbid AF support their inclusion in ongoing randomised trials of oral anticoagulants (NCT03996772, NCT03950076, NCT02565693, NCT03186729, NCT03243175, NCT03153150) and scrutiny of the subgroup with lobar ICH by data monitoring committees.

## Data sharing

Written requests for access to the data reported in this paper will be considered by RA-SS and PMR and a decision made about the appropriateness of the use of data. If the use is appropriate, a data sharing agreement will be put in place before a fully de-identified version of the dataset used for analysis with individual participant data is made available.

## Declaration of interests

TJM reports grants from the British Heart Foundation clinical research training fellowship outside the submitted work. MAR and JJML report grants from The Wellcome Trust during the conduct of the study. RA-SS reports grants from the Medical Research Council during the conduct of the study. All other authors declare no competing interests.
