# Correction to *Lancet Neurol* 2021; 20: 437–47

**DOI:** 10.1016/S1474-4422(21)00185-X

**Published:** 2021-08

*Li L, Poon MTC, Samarasekera NE, et al. Risks of recurrent stroke and all serious vascular events after spontaneous intracerebral haemorrhage: pooled analyses of two population-based studies.* Lancet Neurol *2021;* 20: *437–47*—The appendix of this Article has been corrected as of June 9, 2021.

