# Supplementary material for: Risks of recurrent stroke and all serious vascular events after spontaneous intracerebral haemorrhage: pooled analyses of two population-based studies
Source: Lancet Neurol. 2021 Jun;20(6):437–47. doi: 10.1016/S1474-4422(21)00075-2 (PMC8134058; doi:10.1016/S1474-4422(21)00075-2)
Supplement: Supplementary appendix [file mmc1.pdf]

THE LANCET
Neurology

Supplementary appendix

This appendix formed part of the original submission and has been peer reviewed. We post it as supplied by the authors.

Supplement to: Li L, Poon MTC, Samarasekera NE, et al. Risks of recurrent stroke and all serious vascular events after spontaneous intracerebral haemorrhage: pooled analyses of two population-based studies. *Lancet Neurol* 2021; **20:** 437–47.

Risks of recurrent stroke and all serious vascular events after spontaneous intracerebral haemorrhage: pooled analyses of two population-based studies

Web appendix	Title	Page
1	Search strategy	2
2	Characteristics of all patients with first-in-study-period ICH at diagnosis and hospital discharge in OXVASC (2002-2018) vs. LATCH (2010-2013)	3
3	Characteristics of all OXVASC (2002-2018) patients with first-ever ICH at diagnosis and hospital discharge stratified by ICH location	4
4	Characteristics of all LATCH (2010-2013) patients with first-ever ICH at diagnosis and hospital discharge stratified by ICH location	5
5	Characteristics of all patients with first-ever intracerebral haemorrhage at diagnosis and hospital discharge, stratified by co-morbid atrial fibrillation	6
6	Details of recurrent intracerebral haemorrhage (ICH) and ischaemic stroke (IS) stratified by the location of the index ICH in OXVASC and LATCH	7
7	Absolute event rate (per 100 patient years) of first recurrent ICH, ischaemic stroke, or serious vascular event in patients with ICH stratified by ICH location and co-morbid atrial fibrillation (AF) during 5-year follow-up	8
8	Cox regression analyses of the risks of first recurrent ICH, or ischaemic stroke in lobar vs. non-lobar ICH, AF vs. no AF, premorbid use of antithrombotic drugs vs. none adjusting for clustering	9
9	Risks of first recurrent ICH, ischaemic stroke or any serious vascular event in lobar vs. non-lobar ICH using the competing risk model (death as competing risk)	10
10	Risk of first recurrent ICH, ischaemic stroke or any serious vascular event after lobar vs. non-lobar ICH using the competing risk model (death as competing risk) vs. the Kaplan-Meier model	11
11	PRISMA flow diagram	12
12	Characteristics of studies that reported risks of recurrent ICH or ischaemic stroke, stratified by ICH location	13
13	Time-course of first recurrent ICH, ischaemic stroke or any serious vascular event after lobar vs. non-lobar ICH stratified by premorbid use of antithrombotic drugs	14
14	Risks of first recurrent ICH, ischaemic stroke or any serious vascular event with vs. without co-morbid AF using the competing risk model (death as competing risk)	15
15	Absolute event rate (per 100 patient-years) of first recurrent ICH, ischaemic stroke or serious vascular event in the pooled cohort stratified by co-morbid AF and occlusive vascular disease	16
16	Absolute event rate (per 100 patient-years) of first recurrent ICH, ischaemic stroke or serious vascular event in the pooled cohort stratified by co-morbid AF and occlusive vascular disease using 5-year follow-up data	17
17	Absolute event rate (per 100 patient years) of first recurrent ICH, ischaemic stroke or any serious vascular event in patients with ICH stratified by co-morbid AF and ICH location	18
18	Absolute event rate (per 100 patient years) of first recurrent ICH, ischaemic stroke or any serious vascular event in patients with ICH stratified by co-morbid AF and ICH location using 5-year follow-up data	19
19	Comparison of absolute event rates (per 100 person-years) of outcomes in the RESTART trial and the pooled cohort of OXVASC and LATCH using 5-year follow-up	20
20	Comparison of the baseline characteristics and event rates in LATCH, OXVASC and RESTART (avoid antiplatelet therapy) using all follow-up data	21
21	Comparison of the baseline characteristics and event rates in LATCH, OXVASC and RESTART (avoid antiplatelet therapy) using 5-year follow-up	22
22	Absolute event rate (per 100 person-years) in OXVASC, LATCH, and RESTART stratified by ICH location using all follow-up data	23
23	Absolute event rate (per 100 person-years) in OXVASC, LATCH, and RESTART stratified by ICH location using 5-year follow-up data	24

Web appendix 1 Search strategy

1. exp cerebral haemorrhage/or exp intracranial haemorrhage, hypertensive/
2. (intracerebral adj1 h?emrrhag*) or (h?emorrhag* adj1 stroke) or (intracranial adj1 h?emorrhage)
3. (intracerebral adj1 bleed*) or (intracranial adj1 bleed*)
4. Recurrence/
5. recurrent stroke or recurrent h?emorrhagi* stroke or recurrent intracerebral h?emorrhage or recurrent intracerebral bleed*
6. 1 or 2 or 3
7. 4 or 5
8. 6 and 7
9. Limit 8 to English language
10. Limit 9 to “all adult (19 plus years)”

Web appendix 2 Characteristics of all patients with first-in-study-period ICH at diagnosis and hospital discharge in OXVASC (2002-2018) vs. LATCH (2010-2013)

	OXVASC (n=255)	LATCH‡ (n=419)	p
Age (mean/SD)	76/13	75/12	0.25
Male sex	126 (49.4)	194 (46.3)	0.43
NIHSS (median, IQR)**	8 (4-16)	n/a	n/a
Location - lobar	109 (43.1)	208 (49.6)	0.10
**Medical history**			
Previous occlusive vascular disease*	83 (32.5)	134 (32.1)	0.90
Hypertension	157 (61.6)	272 (64.9)	0.36
Diabetes	36 (14.1)	47 (11.2)	0.27
Hyperlipidaemia	59 (23.1)	75 (17.9)	0.10
Atrial fibrillation	57 (22.4)	90 (21.5)	0.80
Current smoking†	24 (9.7)	92 (22.1)	<0.0001
**Medication before ICH**			
Antithrombotic drugs†	122 (47.8)	222 (53.0)	0.20
Anticoagulant drugs	54 (21.2)	56 (13.4)	0.008
Antiplatelet drugs	73 (28.6)	177 (42.2)	0.0004
Antihypertensive drugs	127 (49.8)	202 (48.2)	0.69
Statins	66 (25.9)	156 (40.1)	0.0002
**Medication at hospital/clinic discharge#**	(n=143)	(n=235)	
Antithrombotic drugs††	8 (5.6)	14 (6.0)	0.88
Anticoagulant drugs	1 (0.7)	6 (2.6)	0.20
Antiplatelet drugs	7 (4.9)	9 (3.8)	0.62
Antihypertensive drugs	104 (72.7)	125 (53.2)	0.0002

Numbers are presented as n (%) unless otherwise stated. * Previous occlusive vascular disease included any history of transient ischaemic attack, stroke, myocardial infarction or peripheral vascular disease. **data missing for n=6 in OXVASC and data not available in LATCH. ‡missing data in LATCH: TIA/stroke (n=1), MI/PVD (n=1), hypertension (n=1), hyperlipidaemia (n=1), AF (n=1), smoking (n=2), statins (n=30). † n=16 on both anticoagulant and antiplatelet drugs. †† n=1 patients on both anticoagulant and antiplatelet drugs. #excluding patients who died before discharge.

Web appendix 3 Characteristics of all OXVASC (2002-2018) patients with first-ever ICH at diagnosis and hospital discharge stratified by ICH location

	**Total (n=255)***	**Lobar ICH (n=109)**	**Non-lobar ICH (n=144)**	**p**
Age (mean/SD)	75.5/13.1	76.4/12.0	74.8/13.8	0.34
Male sex	126 (49.4)	50 (45.9)	76 (52.8)	0.28
**Diagnostic methods**				
CT alone	214 (83.9)	87 (79.8)	125 (86.8)	0.38
MRI alone	4 (1.6)	3 (2.8)	1 (0.7)	
CT and MRI	31 (12.2)	16 (14.7)	15 (10.4)	
Autopsy	6 (2.4)	3 (2.8)	3 (2.1)	
**NIHSS** (median, IQR)**	8 (4-16)	9 (4-17)	8 (4-15)	0.94
**Disabling or fatal**#	199 (78.0)	85 (78.0)	112 (77.8)	0.97
**Blood pressure (mmHg) at onset (mean/SD)**				
Systolic blood pressure	181	170	189	0.0001
Diastolic blood pressure	92	88	94	0.03
**Medical history**				
Previous transient ischaemic attack or stroke	72 (28.2)	39 (35.8)	33 (22.9)	0.03
Myocardial infarction or peripheral vascular disease	21 (8.3)	15 (13.8)	6 (4.2)	0.006
Hypertension	157 (61.6)	58 (53.2)	98 (68.1)	0.02
Diabetes	36 (14.1)	14 (12.8)	22 (15.3)	0.58
Hyperlipidaemia	59 (23.1)	24 (22.0)	35 (24.3)	0.67
Atrial fibrillation	57 (22.4)	24 (22.0)	33 (22.9)	0.87
Current smoker‡	24 (9.7)	10 (9.5)	14 (9.9)	0.92
**Medication before ICH**				
Antithrombotic drugs†	122 (47.8)	55 (50.5)	66 (45.8)	0.47
Anticoagulant drugs	54 (21.2)	27 (24.8)	27 (18.8)	0.25
Antiplatelet drugs	73 (28.6)	30 (27.5)	42 (29.2)	0.77
Antihypertensive drugs	127 (49.8)	49 (45.0)	77 (53.5)	0.18
Statins	66 (25.9)	36 (33.0)	29 (20.1)	0.02
**Medication at hospital/clinic discharge**#	(n=143)	(n=60)	(n=83)	
Antithrombotic drugs	8 (5.6)	6 (10.0)	2 (2.4)	0.05
Anticoagulant drugs	1 (0.7)	1 (1.7)	0 (0)	0.24
Antiplatelet drugs	7 (4.9)	5 (8.3)	2 (2.4)	0.11
Antihypertensive drugs	104 (72.7)	34 (56.7)	70 (84.3)	0.0002
**Discharge location**	(n=143)	(n=60)	(n=83)	0.70
Home	67 (46.9)	28 (46.7)	39 (47.0)	
Community hospital/rehabilitation	71 (49.7)	29 (48.3)	42 (50.6)	
Nursing home/carer/sheltered accommodation	5 (3.5)	3 (5.0)	2 (2.4)	

Numbers are presented as n (%) unless otherwise stated. *n=2 with unknown location due to out-of-area death and no imaging was not accessible; **data missing for n=6. ‡data missing for n=7. # defined as modified Rankin Scale 3 or above. † n=5 patients on both anticoagulant and antiplatelet drugs. #excluding patients that died before discharge.

Web appendix 4 Characteristics of all LATCH (2010-2013) patients with first-ever ICH at diagnosis and hospital discharge stratified by ICH location

	Total‡ (n=419)	Lobar ICH (n=208)	Non-lobar ICH (n=211)	p
Age (mean/SD)	74.3/12.4	75.0/11.4	73.6/13.2	0.26
Male sex	194 (46.3)	89 (42.8)	105 (49.8)	0.15
**Diagnostic methods**				
CT	404 (96.4)	199 (95.7)	205 (97.2)	
MRI	10 (2.4)	6 (2.9)	4 (1.9)	
Autopsy	5 (1.2)	3 (1.4)	2 (0.9)	
**ICH volume (cm^3; median/IQR)***		38 (13-87)	12 (4-31)	
**Medical history**				
Previous transient ischaemic attack or stroke	97 (23.2)	39 (18.8)	58 (27.5)	0.06
Myocardial infarction or peripheral vascular disease	58 (13.9)	25 (12.0)	33 (15.7)	0.28
Hypertension	272 (65.1)	120 (57.7)	152 (72.4)	0.002
Diabetes	47 (11.2)	23 (11.1)	24 (11.4)	0.92
Hyperlipidaemia	75 (17.9)	34 (16.4)	41 (19.5)	0.40
Atrial fibrillation	90 (21.5)	39 (18.8)	51 (24.3)	0.17
Current smoker	92 (22.1)	44 (21.4)	48 (22.8)	0.73
**Medication before ICH**				
Antithrombotic drugs†	222 (53.0)	117 (56.3)	105 (49.8)	0.18
Anticoagulant drugs	56 (13.4)	25 (12.0)	31 (14.7)	0.42
Antiplatelet drugs	177 (42.2)	98 (47.1)	79 (37.4)	0.05
Antihypertensive drugs	202 (48.2)	93 (44.7)	109 (51.7)	0.16
Statins	156 (40.1)	81 (41.5)	75 (38.7)	0.56
**Medication at hospital/clinic discharge#**	(n=235)	(n=122)	(n=113)	
Antithrombotic drugs††	14 (6.0)	6 (4.9)	8 (7.1)	0.48
Anticoagulant drugs	6 (2.6)	4 (3.3)	2 (1.8)	0.46
Antiplatelet drugs	9 (3.8)	3 (2.5)	6 (5.3)	0.26
Antihypertensive drugs	125 (53.2)	55 (45.1)	70 (61.9)	0.01

Numbers are presented as n (%) unless otherwise stated.*ICH volume calculated using ABC/2 method based on CT images. Missing for patients diagnosed by MRI or at autopsy (N=15). ‡missing data: TIA/stroke (n=1), MI/PVD (n=1), hypertension (n=1), hyperlipidaemia (n=1), AF (n=1), smoking (n=2), statins (n=30). #excluding patients that died prior to discharge. † n=11 patients on both anticoagulant and antiplatelet drugs. †† n=1 patient on both anticoagulant and antiplatelet drugs.

Web appendix 5 Characteristics of all patients with first-ever intracerebral haemorrhage at diagnosis and hospital discharge, stratified by co-morbid atrial fibrillation

	Total (n=673) ‡	AF (n=147)	No AF (n=526)	p
Mean age, y (SD)	74.7 (12.6)	79.8 (9.7)	73.3 (13.0)	<0.0001
Male sex	320 (47.5)	73 (49.7)	247 (47.0)	0.56
**ICH location****				
Lobar	317 (47.2)	63 (42.9)	254 (48.5)	0.23
Non-lobar	354 (52.8)	84 (57.1)	270 (51.5)	
**Medical history**				
Previous occlusive vascular disease*	218 (32.4)	74 (50.3)	144 (27.4)	<0.0001
Hypertension	428 (63.6)	129 (87.8)	299 (56.8)	<0.0001
Diabetes	83 (12.3)	26 (17.7)	57 (10.8)	0.03
Hyperlipidaemia	134 (19.9)	42 (28.6)	92 (17.5)	0.003
Current smoker	116 (17.5)	13 (9.0)	103 (19.8)	0.002
**Medication before ICH**				
Antithrombotics†	344 (51.1)	130 (88.4)	214 (40.7)	<0.0001
Anticoagulant drugs	110 (16.3)	84 (57.1)	26 (4.9)	<0.0001
Antiplatelet drugs	250 (37.1)	58 (39.5)	192 (36.5)	0.51
Antihypertensive drugs	329 (48.9)	119 (81.0)	210 (39.9)	<0.0001
Statins	222 (34.5)	68 (46.3)	154 (31.0)	0.001
**Medication at hospital/clinic discharge#**	(n=378)	(n=84)	(n=294)	
Antithrombotic drugs††	22 (5.8)	14 (16.7)	8 (2.7)	<0.0001
Anticoagulant drugs	7 (1.9)	5 (6.0)	2 (0.7)	0.002
Antiplatelet drugs	16 (4.2)	10 (11.9)	6 (2.0)	<0.0001
Antihypertensive drugs	229 (60.6)	68 (81.0)	161 (54.8)	<0.0001

Numbers are presented as n (%) unless otherwise stated. ICH=intracerebral haemorrhage. AF=atrial fibrillation. ‡missing data in LATCH: TIA/stroke (n=1), MI/PVD (n=1), hypertension (n=1), hyperlipidaemia (n=1), AF (n=1), smoking (n=2), statins (n=30); missing data in OXVASC: smoking (n=8).**n=2 with unknown location due to out-of-area death and no imaging was not accessible. *previous occlusive vascular disease included any history of transient ischaemic attack, stroke, myocardial infarction or peripheral vascular disease† n=16 on both anticoagulant and antiplatelet drugs; #excluding patients that died prior to discharge. †† n=1 on both anticoagulant and antiplatelet drugs.

Web appendix 6 Details of recurrent intracerebral haemorrhage (ICH) and ischaemic stroke (IS) stratified by the location of the index ICH in OXVASC and LATCH

	Recurrent ICH:		IS:	
	**Index lobar**	**Index Non-lobar**	**Index lobar**	**Index Non-lobar**
**OXVASC:**	**(n=11)**	**(n=4)**	**(n=4)**	**(n=3)**
**Location of recurrent event**				
Lobar	11 (100.0%)	2 (50.0%)	NA	NA
Non-lobar	0 (0)	2 (50.0%)	NA	NA
**Severity of recurrent event**				
Fatal	4 (36.4%)	2 (50.0%)	1 (25.0%)	1 (33.3%)
Disabling	6 (54.5%)	2 (50.0%)	2 (50.0%)	1 (33.3%)
Non-disabling	1 (9.1%)	0 (0)	1 (25.0%)	1 (33.3%)
**Use of antithrombotics before recurrent event**				
Never on antithrombotics	4 (36.4%)	4 (100.0%)	0 (0)	1 (33.3%)
Antiplatelets stopped & not restarted after index ICH	5 (45.5%)	0 (0)	1 (25.0%)	0 (0)
Anticoagulants stopped & not restarted after index ICH	1 (9.1%)	0 (0)	2 (50.0%)	2 (66.7%)
Antithrombotics restarted after index ICH	1 (9.1%)	0 (0)	1 (25.0%)	0 (0)
**Use of antihypertensives before recurrent event**	3 (27.3%)	3 (75.0%)	3 (75.0%)	3 (100.0%)
**Mean follow-up blood pressure ≤130/80mmHg**	6 (75.0%)*	1 (33.3%)*	1 (25.0%)	1 (50.0%)*
**LATCH:**	**(n=22)****	**(n=9)**	**(n=8)**	**(n=10)**
**Location of recurrent event**				
Lobar	21 (100%)	3 (33.3%)	NA	NA
Non-lobar	0 (0)	6 (66.7%)	NA	NA
**Severity of recurrent event**				
Fatal	6 (27.3%)	3 (33.3%)	1 (1.3%)	2 (20%)
Non-disabling	16 (72.7%)	6 (66.7%)	7 (8.7%)	8 (80%)
**Use of antithrombotics before recurrent event**				
Never on antithrombotics	11 (50.0%)	4 (44.4%)	3 (37.5%)	4 (40.0%)
Antiplatelets stopped & not restarted after index ICH	7 (31.8%)	1 (11.1%)	2 (25.0%)	3 (30.0%)
Anticoagulants stopped & not restarted after index ICH	1 (4.5%)	0 (0)	0 (0)	1 (10.0%)
Antithrombotics restarted after index ICH	3 (13.6%)	2 (22.2%)	2 (25.0%)	1 (10.0%)
New antithrombotics initiated during follow-up	0 (0)	2 (22.2%)	1 (12.5%)	1 (10.0%)
**Use of antihypertensives before recurrent event**	11 (50.0%)	6 (66.7%)	6 (75.0%)	7 (70.0%)
**Mean follow-up blood pressure ≤130/80mmHg**	4 (33.3%)**	2 (28.5%)**	0 (0)**	0 (0)**

NA=not applied, ICH=intracerebral haemorrhage, IS=ischaemic stroke. *follow-up blood pressure available for 8/11, 3/4, 2/3 respectively for recurrent ICH after index lobar, non-lobar ICH and recurrent IS after non-lobar ICH. ** follow-up blood pressure available for 12/22, 7/9, 7/8, and 9/10 respectively for recurrent events after index lobar and non-lobar ICH. One patient with recurrent ICH was identified at post-mortem, therefore no location information available.
